# Effect of Cu Substrate Roughness and Sn Layer Thickness on Whisker Development from Sn Thin-Films

**DOI:** 10.3390/ma12213609

**Published:** 2019-11-03

**Authors:** Balázs Illés, Tamás Hurtony, Olivér Krammer, Bálint Medgyes, Karel Dušek, David Bušek

**Affiliations:** 1 Department of Electronics Technology, Budapest University of Technology and Economics, 1111 Budapest, Hungary; hurtony@ett.bme.hu (T.H.); krammer@ett.bme.hu (O.K.); medgyes@ett.bme.hu (B.M.); 2 Department of Electrotechnology, Czech Technical University in Prague, 166 27 Prague, Czech Republic; dusekk1@fel.cvut.cz (K.D.); busekd1@fel.cvut.cz (D.B.)

**Keywords:** whisker, thin-film, surface roughness, intermetallic layer, FIB

## Abstract

The effect of copper substrate roughness and tin layer thickness were investigated on whisker development in the case of Sn thin-films. Sn was vacuum-evaporated onto both unpolished and mechanically polished Cu substrates with 1 µm and 2 μm average layer thicknesses. The samples were stored in room conditions for 60 days. The considerable stress—developed by the rapid intermetallic layer formation—resulted in intensive whisker formation, even in some days after the layer deposition. The developed whiskers and the layer structure underneath them were investigated with both scanning electron microscopy and ion microscopy. The Sn thin-film deposited onto unpolished Cu substrate produced less but longer whiskers than that deposited onto polished Cu substrate. This phenomenon might be explained by the dependence of IML formation on the surface roughness of substrates. The formation of IML wedges is more likely on rougher Cu substrates than on polished ones. Furthermore, it was found that with the decrease of layer thickness, the development of nodule type whiskers increases due to the easier diffusion of other atoms into the whisker bodies.

## 1. Introduction 

Tin whiskers are mono- or polycrystalline surface eruptions [1]. They can develop from Sn surface coatings [2], solder joints [3] or thin-film layers [4]. Their usual dimensions are within 1–10 µm in thickness, and within 10–1000 µm in length, depending on the type of the whisker: filament, nodule or hillock [5]. Whisker development denotes serious reliability risk in microelectronic devices because the longer whiskers (>100 µm) can form short circuits between component leads. A recent trend in microelectronics is to use low silver content micro-alloyed solders (in which the tin content can reach 98–99 wt%) and pure tin surface finishes. In these material systems, whisker formation is even more likely than it was at tradition SnAgCu and SnCu alloys [3]. Sn whisker development is always induced by stress arising inside the Sn layer. Actually, the whisker development is the stress release of the Sn layer against internal stresses, which can originate from direct mechanical load (by test needles, connectors, etc.) [6], from residual stress during deposition of the Sn layer [2], from volumetric expansion inside the layer structure (like oxide formation, intermetallic layer growth) [7], and from thermomechanical effects [8]. 

The physical properties of the Sn layers affect their susceptibility also for whisker development. The main influencing properties of the layer are the size, shape, and crystallographic structure of the grains (mono- or polycrystalline and the orientation) [9,10], and the layer thickness [11]. In a fine grain structure (when the average grain size is smaller than 500 nm) the diffusivity is higher due to many grain boundaries [12], therefore the growth of the Intermetallic Layer (IML) is faster [13,14]. The more intensive growth of the IML layer causes more internal stress by volumetric expansion. However, lots of grain boundaries (in a fine grain structure) helps the Sn layer to relax the stress. A similar effect is related to the shape of Sn grains. Globular/horizontal grain structure has better stress relaxation ability than that of the columnar grain structure [15]. Furthermore, Yu et al. [16] found that the formation of wedge type intermetallics (which yields significant stress on the neighbouring grain) is more likely between columnar grains than between horizontal ones. 

The relation between the whisker development and the crystallographic structure of Sn grains was also elaborated. Jagtap et al. [17] reported that whisker growth is more likely from the Sn grains with lower indices, like <100> or near-<100>, than from higher ones, like <321>. Eckold et al. [18] showed similar results, that the <211> orientation is a preferred orientation because this orientation ensures lower corrosion propensity than the <220> or <321>. Zhang et al. [19] reported that whisker susceptibility is generally low in Sn layers with multi-peak texture (like <112> <101> <103>). The crystallographic structure of Sn layers can affect the IML formation as well since the IML develops the most towards the c-axis direction of Sn grains [13]. The relationship between the Sn layer thickness and the susceptibility of whisker development is straightforward. Thick Sn layers (>10 µm) develops slower and fewer amounts of Sn whiskers than thin Sn layers (<5 µm). The reason is that more time is required for the intermetallic grains to reach the Sn grains at the surface of the Sn layer, and to create a stressed cell [20]. The layer thickness of vacuum evaporated Sn layer is usually between 0.1–2 µm and it contains globular grains in micron-scale. In the case of soldering technologies, the microelectronic devices mostly use much thicker Sn layers than the micron-scale, but the Sn or Cu-Sn film layers are used as contact layer for Cu bonding [21], and as anode layer for lithium-ion batteries [22]. 

Former research works reported that Sn whisker growth from thin-films is initiated by the residual stress in the thin layer which can be controlled by the pressure during deposition. Bozack et al. [23] found Sn whiskers on 1–5 µm thick, sputtered Sn thin-films on a brass substrate, and they thought that using a relatively low background pressure (1 mTorr), created stress in the thin-film. Chen and Shih [24] studied whisker growth on a 1 µm thick evaporated Sn layer on a Cu laminate at room conditions. The Sn whisker development was explained by the release of residual stress created by the evaporation (or electroplating) process. Cheng et al. [25] prepared 1 µm evaporated Sn thin-film on bent silicon substrates, and kept the samples at 180 °C in vacuum. They stated that the whisker growth was caused by two different types of mass transport: the grain boundary diffusion and the interface fluid transport. Crandall [26] investigated the effect of electropolishing the brass substrate in the case of ultra-thin (<150 nm) films deposited by magnetron sputtering. She found more whiskers on the polished samples, but the root causes have not been discussed.

According to our literature survey, only a few researches study whisker growth from regular Sn thin-films evaporated onto the Cu substrate. Therefore, the aim of our research was to find if there is a significant effect of the Cu substrate roughness and the layer thickness of Sn thin-film on whisker development. 

## 2. Materials and Methods

Electron Beam–Physical Vapour Deposition (EB-PVD) technology was used for sample preparation. Pure Sn (99.99%) was vacuum evaporated (Balzers BA 510) onto Cu substrates. Before the evaporation, the following preparatory steps were carried out on the Cu substrates: relaxing at 200 °C for 3 h; chemical etching to remove the surface oxides; cleaning in isopropyl alcohol; and ion bombarding to neutralize the surface. Evaporation happened with 100 mA cathode heating current and 7 kV acceleration voltage in high vacuum (10^−3^ Pa). Three different sample types were fabricated, according to the variation of Cu substrate roughness and the Sn layer thickness (Table 1). The polished samples had been ground manually in crosshatched pattern with consecutive series of P600, P1200 and P4000 of SIC grinding papers. Then two-step polishing process was applied with 3 and 1 μm diamond suspension on buffer wheel. The surface roughness of the Cu substrates was measured by Alpha-Step 500 surface profiler, it was 0.423 ± 0.037 μm (with grainlines scratches) and 0.187 ± 0.015 μm (without oriented scratches) on the unpolished and polished substrates, respectively. In the case of S2 and S3 samples, the same piece of substrate was used, it was halved before the evaporation of the Sn layer.

**Figure 1:**
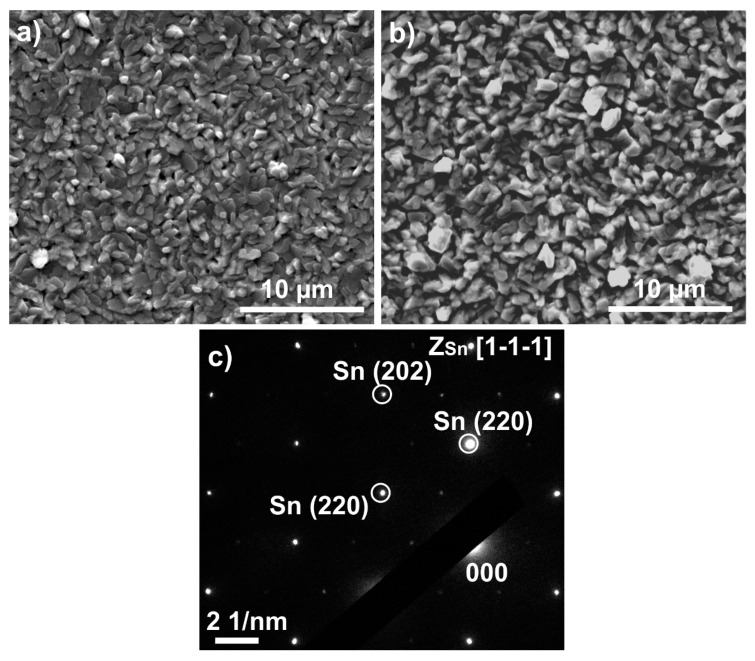
(**a**) Surface morphology and grain orientation of Sn thin-film layers right after the layer deposition, 1 µm Sn thickness; (**b**) 2 µm Sn thickness; (**c**) general diffraction pattern.

Figure 1a,b shows the SEM micrograph about the surface of the Sn layers. The layer thickness affects the grain size, which is 0.5–1 μm and 1–2 μm in the case of the 1 µm and 2 μm thick Sn layers respectively. The grain orientation of the Sn thin-film was determined by Selected Area Electron Diffraction (SAED) technique, and it was found to be generally <111>, Figure 1c [27]. 

Samples were stored at room conditions (22 ± 1 °C/50 ± 5 RH%) in a climatized laboratory for up to 60 days. Whisker development was monitored within regular intervals by a Scanning Electron Microscope SEM (FEI Inspect S50) with 20 kV of accelerating voltage. An automatic image processing method was used on the SEM micrographs to determine grains sizes, whisker densities, and lengths. The method includes an adaptive binarization algorithm for separating whiskers from the background. The binarization is carried out according to the mean intercept length of the separated structures [28]. The whisker density was calculated in pcs./mm^2^. The length of the whisker was calculated according to the major axis of an overlaying ellipse placed on the whisker. Top-view images were investigated and measured about the whiskers because, from the reliability aspect of electronic devices, the orthogonal projection of whisker’s length is critical from the aspect if whiskers can form a short circuit between adjacent component leads or not. All points of the statistics were calculated from 20 SEM micrographs. Cross-sections were prepared by a Dual-Beam Focused Ion Beam FIB (Thermo Scientific Scios 2) to investigate the layer structure of the samples. Micrographs were prepared by a FIB Scanning Ion Microscopy (FIB-SIM) with Ga ion source and 30 kV of accelerating voltage. 

## 3. Results

**Figure 2:**
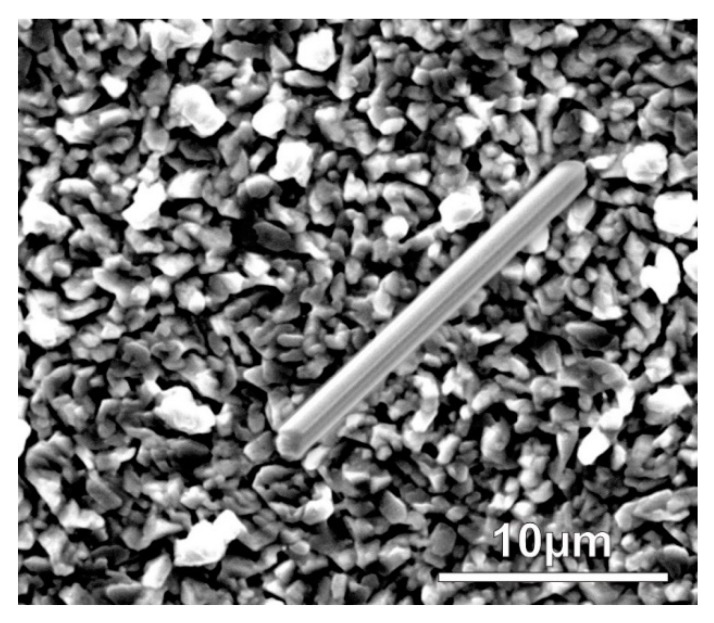
One of the first whiskers (17 µm long) on the sample S2 at 3 days after the layer deposition.

**Figure 3:**
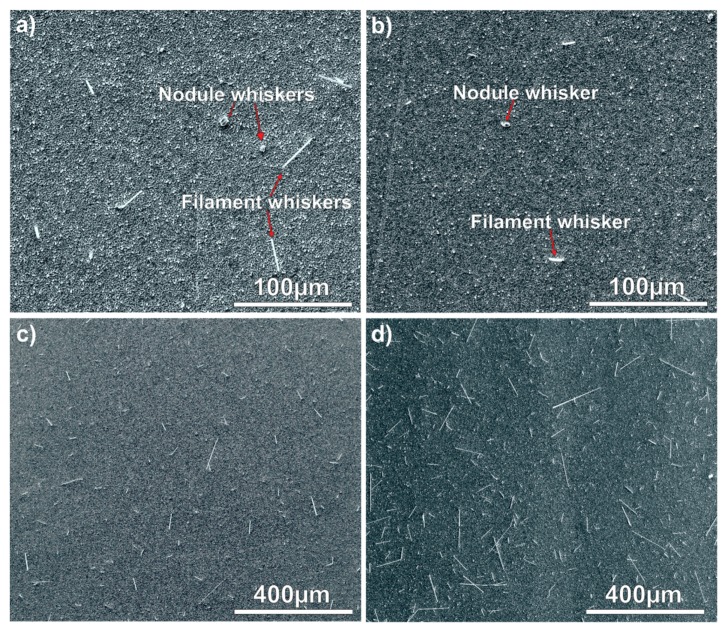
Whiskers on the samples: (**a**) filament- and nodule-type whiskers on sample S2 after 7 days; (**b**) filament- and nodule-type whiskers on sample S3 after 7 days; (**c**) numerous 50–100 µm long filament whiskers on sample S2 after 60 days; (**d**) numerous filament (50–200 µm long) and nodule-type whiskers on sample S3 after 60 days.

The large internal stress caused by the formation of IML initiated the whisker growth right after the layer deposition. Some eruptions (and hillocks) appeared even in 1 day after the layer deposition, and the first filament whiskers were found already after 3 days on each sample type (Figure 2). However, some nodule type whiskers were also found at the 7th days, but most of the developed whiskers were still filament type (Figure 3a,b). The thickness of the filament-type whiskers was usually the same as the average grain size ~0.5–2 µm (Figure 1), while the nodule-type whiskers were usually much thicker, even ~5–10 µm thick (Figure 3a,b).

In the case of the 2 μm layer thickness (samples S1 and S2), the number of nodule-type whiskers did not increase during the research; almost only filament whiskers were found (Figure 3c). In the case of samples S3, the nodule-type whiskers started developing in 7 days after the layer deposition, and until the end of the research, most of the whiskers were nodule-type (Figure 3d). During the 60 days of the research, numerous tin whiskers developed on all sample types. The filament type whiskers reached even hundreds of micrometers (Figure 3c,d).

**Figure 4:**
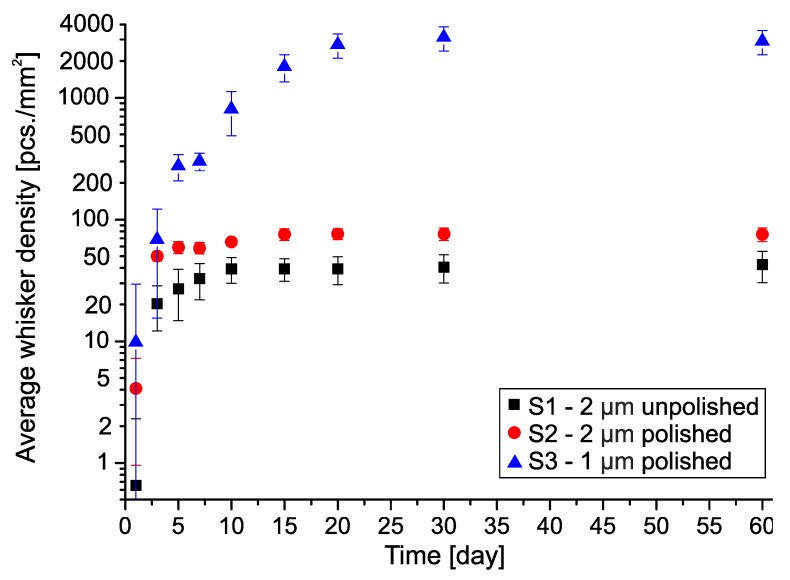
The average density of Sn whiskers.

During the statistical evaluation, three main parameters of the grown whiskers were determined: the whiskers’ area density, the average length and the maximum length of filament-type whiskers. From reliability aspects, mostly the filament-type whiskers are important, since the nodule-type ones usually not able to cause short circuit failure in microelectronics. According to the first visual inspection of the SEM micrographs, no significant difference was found between the unpolished (S1) and polished (S2) Cu substrates with the same 2 μm Sn layer thickness. However, the results of the statistical evaluation pointed out some considerable differences between these samples. It was similar that the average whisker density increased only up to 10–15 days after the layer deposition, and it reached only 40 pcs./mm^2^ on the samples S1 and 75 pcs./mm^2^ on the samples S2 (Figure 4).

Contrary, the average whisker density increased up to 20–25 days after the layer deposition in the case of the 1 μm thick Sn layer (S3), and the density reached even 2900 pcs./mm^2^. However, the ratio of filament-type whiskers to the nodule-type ones was only 6.7% (~195 pcs./mm^2^). In this aspect, the increase is less significant, only 2.5–5 times larger compared to that in samples with 2 μm thick Sn layer. In one of our previous work about 400 nm thick Sn thin-films, it was found that the whisker density increased until 90 days after the layer deposition, and the area density reached 3900 pcs./mm^2^ [5], so the results are correlating. 

**Figure 5:**
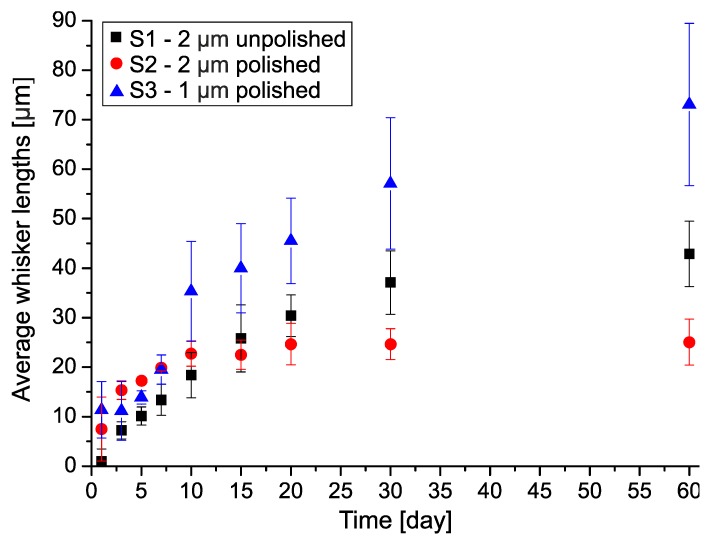
The average length of the filament Sn whiskers.

Like the average whisker density, the average length of filament-type whiskers also showed saturation type characteristics in the case of samples S1 and S2 (Figure 5). The trend changed 15 days after the layer deposition between the samples with 2 μm thick Sn layer (between samples S1 and S2). Longer whiskers developed on the sample S1 than on the sample S2. The average length was 42 μm on sample S1, and 25 μm on the sample S2, 60 days after the layer deposition. In the case of 1 μm Sn layer, the increase of average length did not saturate until 60 days, and it reached 73 μm on average. From this aspect, the differences in whisker lengths between the samples with 1 µm and 2 μm thick Sn layers are smaller than the differences between them in the average densities. (In the case of the nodule-type whiskers, the average length is 7 μm).

**Figure 6:**
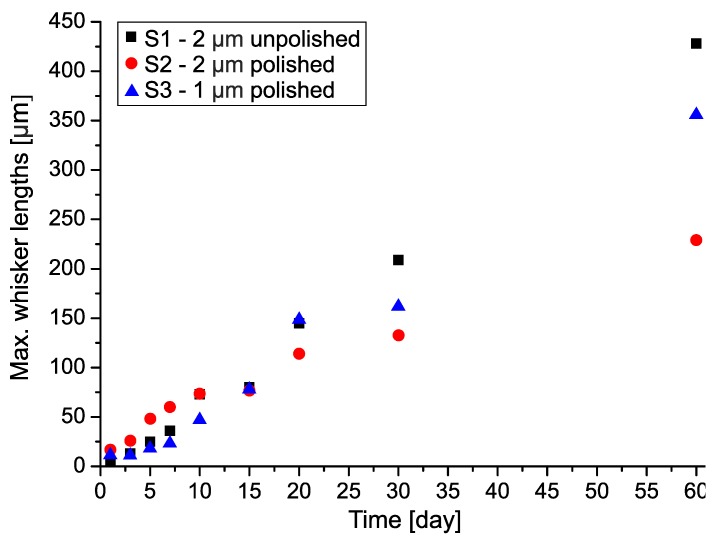
The maximum filament whisker lengths.

The characteristics of the maximal filament-type whisker lengths were much more similar and closer to each other than the previous statistical parameters. The characteristics of the maximal filament whisker length did not show any saturation; the growth rate was linear until the end of the evaluation period (Figure 6). Up to 15 days after the layer deposition, there was no considerable difference between the samples. The longest filament whiskers were around 80 μm on all samples. Later, the difference in maximal length started to increase. Finally, the unpolished Cu substrate with 2 μm thick Sn layer (S1) produced the longest detected filament-type whisker, which was 420 µm. The longest detected Sn whiskers were 200 µm and 355 μm on samples S2 and S3 respectively. In our previous research concerning 400 nm thick Sn layer, the maximum filament whisker lengths were quite similar, 275 μm, 70 days after the layer deposition [5]. Note that the pitch size of recently used fine-pitch components is below 200 μm [29]. Therefore, the vacuum evaporated Sn thin-films can cause considerable reliability risk in microelectronic devices, in spite of the Sn layer thickness or the surface roughness of Cu substrates. 

**Figure 7:**
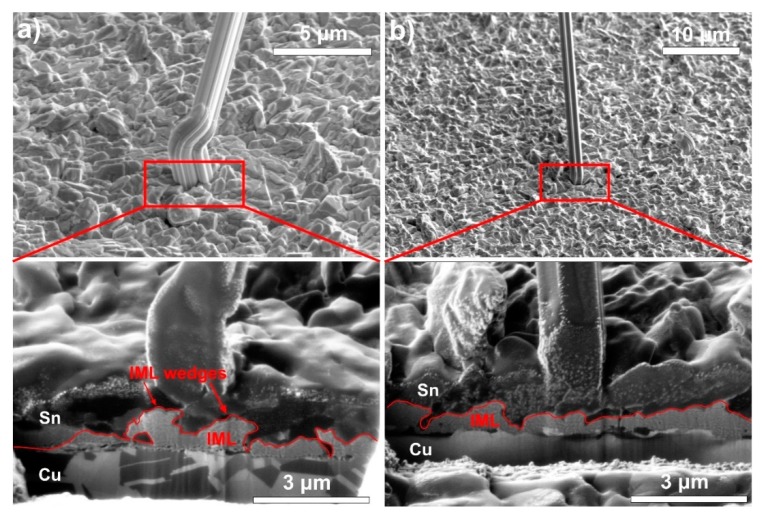
FIB-SIM micrographs of the layer structure under the whiskers 60 days after the layer deposition: (**a**) 2 μm thick Sn thin-film on unpolished Cu substrate; (**b**) 2 μm thick Sn thin-film on polished Cu substrate.

FIB cuts were prepared for investigating the layer structures of samples S1 and S2 (Figure 7). It was found that the vacuum evaporation resulted in a globular Sn grain structure in both cases. The rapid IML layer formation—as the root cause for whisker growth–is evident. 60 days after the layer deposition, the IML already consumed 50% of the 2 μm thick Sn layer. Generally, the IML layer is much more even (smaller differences in the layer thickness) in the case of polished Cu substrates (S2) than in the case of unpolished Cu substrate (S1). Besides, it was found that the formation of IML wedges is more likely on the unpolished Cu substrate (Figure 7a). 

## 4. Discussion 

**Figure 8:**
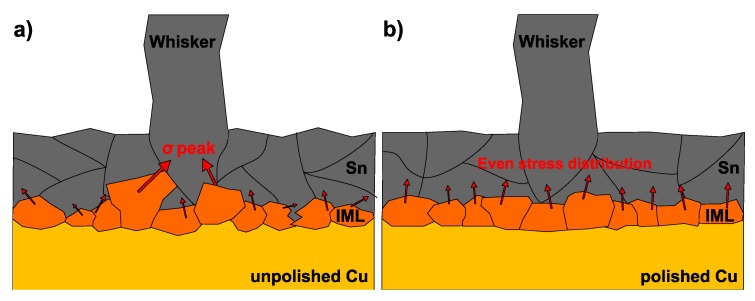
Stress distribution in the Sn layer: (**a**) unpolished Cu substrate with uneven Intermetallic Layer (IML) layer (samples S1); (**b**) polished Cu substrate with even IML layer (S2).

The most important findings of this research are the whisker development differences between the polished and unpolished Cu substrates and the increasing number of nodule-type whiskers with the decrease of Sn layer thickness. The samples with the unpolished Cu substrate (S1) produced less but longer whiskers than the samples with polished Cu substrate (S2). The rougher Cu substrate had higher surface energy, which resulted in intensive diffusion and formation of an uneven IML layer (Figure 7a). Uneven IML areas (with IML wedges) had a larger surface—the Sn grains might be loaded which through—than an even IML layer. Besides, the shape of the IML wedges can concentrate the stress [30]. Kim et al. [31] reported similar results in the case of electroplated Sn layer with ~2 μm thickness, and Zhang et al. [32] related the decreased whisker growth to the evenness IML layer, in the case of chemical Sn layer. Therefore, our hypothesis is that in the case of unpolished Cu substrate (S1), the IML wedges could exhibit stress peaks in some regions of the Sn layer, where very long filament whiskers [5,8] could grow. Nevertheless, the distribution of the stress generated by an uneven IML layer was inhomogeneous (Figure 8a). Generally, it resulted in lower stress levels on the Sn layer, since the different stress components could neutralize each other, and finally it produced fewer whiskers. In comparison, in the case of polished Cu substrate (S2), the stress distribution was homogeneous over an even IML layer (Figure 8b). Generally, it could cause larger stress level on the Sn layer but without considerable stress peaks which could result in more but shorter whiskers.

The effect of the Sn layer thickness on the whisker development is straightforward. Comparing the results of samples S2 and S3 (polishing was used on both substrates), the 1 μm thick Sn thin-film produced 2.5-times more and 1.75-times longer filament-type whiskers than the 2 μm thick Sn thin-film. In addition, the thinner Sn layer developed whiskers for a more extended period than the thicker one. This tendency was continuous with the decrease of the Sn layer thickness according to the results of [5]. The effect of the layer thickness was due to the better mechanical stress relaxation ability of thicker Sn layers. In the case of the 2 μm thick Sn layer (S2), the stress level had a drop at 10–15 days after the layer deposition. After this period, newly developed whiskers were rarely found, only the length of the already developed ones increased (Figure 4 and Figure 5). In the case of 1 μm thick Sn layer (S3), the drop of the stress level occurred only at 20–25 days after the layer deposition (Figure 4 and Figure 5).

The other considerable difference between the samples with different layer thicknesses was the larger number of nodule-type whiskers on the thinner Sn layers (S3) than on the thicker one (S2). This result agrees to the result of Crandall [26], who found remarkable number of nodule-type whiskers in the case of ultra-thin-film Sn layers. According to the literature, one of the root causes of nodule-type whisker formation is the contaminants inside the whisker. Contaminants can include atoms (like Ag, Cu, Bi, etc.,) from the solder alloy and wirings, which can diffuse into the whisker. These materials could result in the formation of nodule-type whiskers by the twisting of the whisker body [33]. In our case, thicker Sn layers could delay the diffusion of Cu atoms from the Cu substrate into the whisker, and it could delay the formation of nodule-type whiskers. No effect of the Cu substrate roughness was observed in the shape of the whiskers. 

## 5. Conclusions

The effect of Cu substrate roughness and Sn layer thickness were investigated on whisker development in the case of Sn thin-films. The considerable stress—arisen by the rapid intermetallic layer formation—resulted in numerous tin whiskers, even in some days after the layer deposition. The developed filament-type whiskers can cause considerable reliability risk in microelectronic devices. Whisker development differences were found between the polished and unpolished Cu substrates. Sn thin-film on unpolished Cu substrate produced less but longer whisker than that on polished Cu substrate which might be explained by the differences of the IML formation. According to our hypothesis, unpolished Cu substrate produced a non-uniform IML layer with wedge-shaped IML grains, which resulted in rarer but long whiskers at stress peaks of the IML wedges. Polished Cu substrate produced a uniform IML layer with evenly high stress levels on the Sn layer which produced a lot but short filament-type whiskers. The decrease of the layer thickness increased considerably the number of tin whiskers and moderately the average length of them. The amount increase was due to the development of nodule-type whiskers on the thinner Sn thin-film. This can be explained by the easier diffusion of Cu atoms into the whiskers, which can yield in the formation of nodule-type whiskers. Therefore, it is suggested for the microelectronics industry to use polished Cu substrates to decrease the length of the filament whiskers and thicker Sn thin-films to decrease the number of whiskers. Furthermore, an extended set of sample types will be necessary to be able to fully characterize the effect of Cu surface roughness and Sn film layer thickness on whisker on Sn whisker growth.

## Figures and Tables

**Table 1:** Sample types.

Samples	Substrate Preparation	Average Cu Roughness [µm]	Sn Layer Thickness [µm]
S1	Unpolished	0.423 ± 0.037	2
S2	Polished	0.187 ± 0.015	2
S3	Polished	0.187 ± 0.015	1
